# *Echinococcus multilocularis* and other cestodes in red foxes (*Vulpes vulpes*) of northeast Italy, 2012–2018

**DOI:** 10.1186/s13071-020-04520-5

**Published:** 2021-01-07

**Authors:** Carlo Vittorio Citterio, Federica Obber, Karin Trevisiol, Debora Dellamaria, Roberto Celva, Marco Bregoli, Silvia Ormelli, Sofia Sgubin, Paola Bonato, Graziana Da Rold, Patrizia Danesi, Silvia Ravagnan, Stefano Vendrami, Davide Righetti, Andreas Agreiter, Daniele Asson, Andrea Cadamuro, Marco Ianniello, Gioia Capelli

**Affiliations:** 1 Istituto Zooprofilattico Sperimentale delle Venezie, Legnaro (PD), Italy; 2 Provincia di Belluno, Wildlife Management Office, Belluno, Italy; 3 Provincia di Bolzano, Wildlife Management Office, Bolzano, Italy; 4 Provincia di Trento, Wildlife Management Office, Trento, Italy; 5 Regione Friuli Venezia Giulia, Wildlife Management Office, Udine, Italy; 6 Ministry of Health, General Directorate for Animal Health and Veterinary Drugs, Rome, Italy

**Keywords:** *Echinococcus multilocularis*, Alveolar echinococcosis, Cestode, *Vulpes vulpes*, Northeast Italy

## Abstract

**Background:**

*Echinococcus multilocularis* is a small tapeworm affecting wild and domestic carnivores and voles in a typical prey-predator life cycle. In Italy, there has been a focus of *E. multilocularis* since 1997 in the northern Italian Alps, later confirmed in red foxes collected from 2001 to 2005. In this study, we report the results of seven years of monitoring on *E. multilocularis* and other cestodes in foxes and describe the changes that occurred over time and among areas (eco-regions) showing different environmental and ecological features on a large scale.

**Methods:**

Eggs of cestodes were isolated from feces of 2872 foxes with a sedimentation/filtration technique. The cestode species was determined through multiplex PCR, targeting and sequencing ND1 and 12S genes. Analyses were aimed to highlight variations among different eco-regions and trends in prevalence across the study years.

**Results:**

Out of 2872 foxes, 217 (7.55%) samples resulted positive for cestode eggs at coproscopy, with differences of prevalence according to year, sampling area and age class. Eight species of cestodes were identified, with *Taenia crassiceps* (2.65%), *Taenia polyacantha* (1.98%) and *E. multilocularis* (1.04%) as the most represented. The other species, *Mesocestoides litteratus, Taenia krabbei, T. serialis, T. taeniaeformis* and *Dipylidium caninum*, accounted for < 1% altogether. *Echinococcus multilocularis* was identified in foxes from two out of six eco-regions, in 30 fecal samples, accounting for 1.04% within the cestode positives at coproscopy. All *E. multilocularis* isolates came from Bolzano province. Prevalence of cestodes, both collectively and for each of the three most represented species (*T. crassiceps, T. polyacantha* and *E. multilocularis*), varied based on the sampling year, and for *E. multilocularis* an apparent increasing trend across the last few years was evidenced.

**Conclusions:**

Our study confirms the presence of a focus of *E. multilocularis* in red foxes of northeast Italy. Although this focus seems still spatially limited, given its persistence and apparent increasing prevalence through the years, we recommend research to be conducted in the future on the ecological factors that, on a smaller scale, allow this zoonotic species to persist. On the same scale, we recommend a health education campaign to inform on the measures to prevent this zoonosis, targeted at people living in the area, especially hunters, dog owners, forestry workers and other potentially exposed categories.
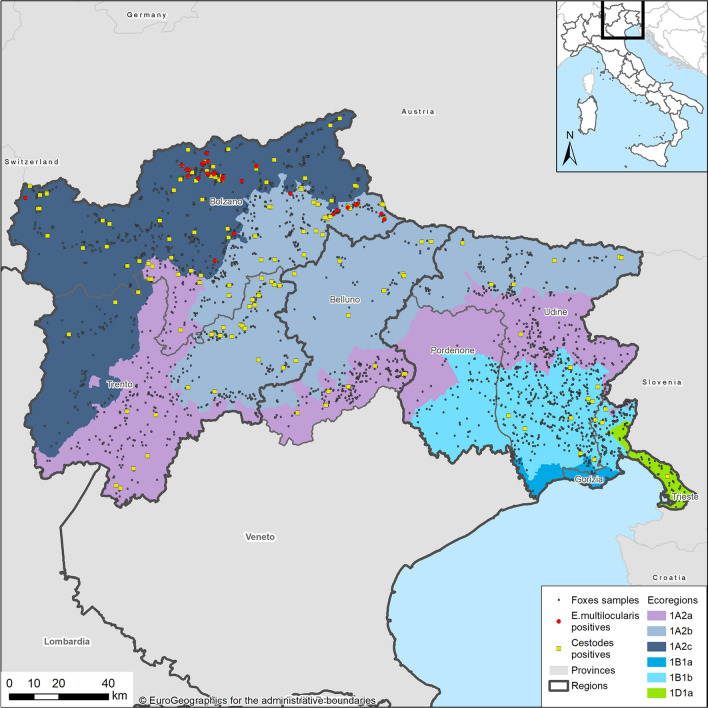

## Background and aim of the study

*Echinococcus multilocularis* (Cestoda, Cyclophillidea, Taeniidae) is a small tapeworm affecting wild and domestic carnivores and voles in a typical prey-predator life cycle [1]. The principal cycle involves the red fox (*Vulpes vulpes*) as the definitive host and small rodents (muskrats and voles) as intermediate hosts. Other carnivores however, such as the Arctic fox (*Vulpes lagopus*), wolf (*Canis lupus*), raccoon dog (*Nyctereutes procyonoides*), golden jackal (*Canis aureus*), dog (*Canis lupus familiaris*) and cat (*Felis catus*), may act as definitive hosts [2–5]. The eggs excreted with the feces by the definitive hosts contaminate the environment and represent the infective stage for the intermediate hosts, including humans. Humans are accidental hosts and may acquire the infection by ingesting contaminated water [6] or fresh fruits, vegetables and mushrooms [7]. In the intermediate hosts, *E. multilocularis* is the causative agent of alveolar echinococcosis, a serious disease that in humans can be fatal if not treated [8]. Indeed, beside rabies, *E. multilocularis* represents the major zoonotic agent transmitted from foxes to humans, and a group of experts ranked it first as a priority infection among food-borne pathogens in Europe [9].

The present study is based on samples from the definitive host, the red fox, which is one of the most common wild carnivores nearly all over Italy, where it is culled for sport hunting or population control purposes, as this species is considered generally abundant and does not show particular conservation issues [10]. It shows a great ecological plasticity and a variable diet, including carnivorous feeding habits relying on ungulates and rodents, but also domestic animals and invertebrates, as well as different food sources as fruits and rubbish [11, 12]. Their high adaptability has allowed red foxes to colonize all the Alps and any environment within this area. Few studies however have addressed local population densities: in northeastern (NE) Italy, which represents our study area, values are reported ranging from 1.77 ± 1.14 individuals/km^2^ in Friuli Venezia Giulia region [13] to 2.11 ± 0.56 individuals/km^2^ in Bolzano province in 2018 (Celva R., personal communication) and 1.34 (pre-reproductive) 3.38 (post-reproductive) in southeastern Belluno province in 2000–2003 (Wildlife Management Office of Belluno Province, personal communication). In this area, during the last 2 decades the alpine fox population recovered from a rabies epizootic, which started in late 2008 and was eradicated by oral fox vaccination in early 2011 [14]. Moreover, since 2006 it has also been affected by consecutive canine distemper virus outbreaks [15]. Although not always consistently among different local guidelines, in NE Italy the fox demographic trends are monitored annually through the analysis of relative abundance indices such as passive surveillance results, den counts, hunting bag sizes and, more recently, spotlight counts performed simultaneously with red deer counts [16].

*Echinococcus multilocularis* in Europe is widely distributed in the temperate and cold areas of northern and eastern Europe; however, reports of the parasite are increasing in southern regions, such as the Balkans, Greece and Italy [4, 17, 18]. Among Member States of Europe, only Finland, Ireland, Malta and the UK have evidence of the continuous absence of this parasite species in their wild and domestic populations of definitive hosts [19]. The EU recognizes that *E. multilocularis* is relevant to some Member States and that to prevent it from spreading there is a need for surveillance and standards for the movement of dogs, cats and ferrets within the Union, such as evidence of anti-cestode treatments [20]. In Italy, *E. multilocularis* has been reported for the first time in red foxes shot from 1997 to 2001 in the northern Italian Alps, close to the Austrian border [21], and it was later confirmed in foxes collected from 2001 to 2005 in the same area [22, 23]. Other studies did not find *E. multilocularis* in central Italy [24, 25], while recently the parasite has been detected in shepherd dogs and wolves in the southwestern Italian Alps, near the French border [26].

In 2011, several years after the first report of *E. multilocularis* in the northern Alps, the Italian Ministry of Health funded a novel monitoring of this cestode in red foxes, starting from the old focus and extending the sampling area.

The main aims of the present study are (i) to report the results of the last 7 years monitoring, describing the changes that occurred over time and among different areas in the prevalence of *E. multilocularis* as well as in the whole cestode community and (ii) based on present and historical data, to resume and collate relevant information on the factors likely to play a key role for the presence of *E. multilocularis* in our focus.

## Methods

### Study area

The study area is located in NE Italy (Wgs84-longitude 10.396302, latitude 45.587717; longitude 13.983849, latitude 47.106623), bordering Austria and Slovenia and covering the territory of seven provinces (Bolzano, Trento, Belluno, Pordenone, Udine, Gorizia and Trieste) for about 2514 km^2^ of mainly alpine landscape. Average altitude is approximately 1230 m a.s.l., ranging from sea level in the southeastern portion (Po River plain) to the highest values (up to 3905 m a.s.l.) in the central and western Dolomitic complexes. Overall monthly mean temperature (recorded from 1970 to 2000) ranges from − 2.16 °C in December to 15.69 °C in June [27].

**Fig. 1:**
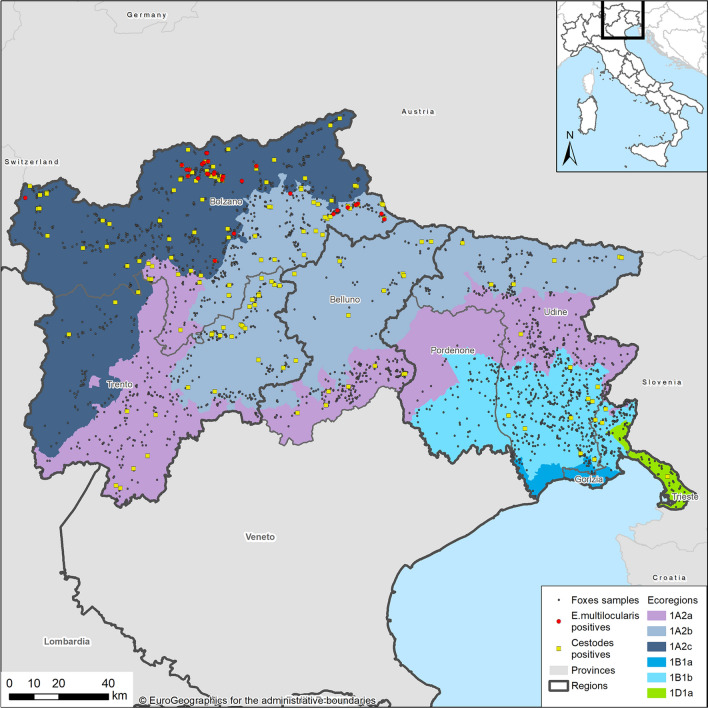
Distribution of cestode-positive and *E. multilocularis*-positive foxes (whole sample) according to province and ecoregions in the northeast of Italy

To present our results in explicitly ecological terms, although on a large scale, we referred to the framework given in Blasi et al. [28], therefore discretizing our study area into six distinct ecoregions (Fig. 1). Spatial discretization is based on potential natural vegetation (PNV), which is an expression of specific underlying climatic, biogeographic, physiographic and hydrographic factors and is considered to be relatively stable in time, providing a reference model on which to monitor the effects of natural dynamics. Ecoregions are briefly described in Table 1 (see [28] for further details).

**Table 1:** Ecoregion characterization. Precipitation and temperature refer to mean annual range between stations

Code	Name	Elevation range (m above sea level)	Precipitation (mm)	Temperature (°C)	Bioclimatic character	Land cover
1A2a	Pre-Alps	45–2609	805–2628	2–14	Temperate semicontinental with subcontinental sectors near the Po River plain	Mainly natural-seminatural areas
1A2b	Dolomiti and Carnia	250–3263	690–2196	4–11	Temperate semicontinental with subcontinental sectors near the pre-Alps	Dominance of natural-seminatural areas
1A2c	Northeastern Alps	200–4049	567–1302	5–12	Temperate semicontinental with widespread oceanic sectors in Alpi Lombarde	Dominance of natural-seminatural areas
1B1a	Lagoon	0–15	581–733	13–14	Temperate subcontinental	Dominance of agricultural areas
1B1b	Central Plain	0–603	701–1346	11–14	Temperate semicontinental with widespread semicontinental-subcontinental sectors	Dominance of agricultural areas

Ecoregion 1D (Italian part of Illyrian Province) was omitted since no reliable information is available

### Sample collection

In the period 2012–2018, we collected fecal samples from red foxes legally hunted or found dead in the provinces listed above, which were given to our laboratories for different purposes (e.g. general diagnostics, rabies surveillance, oral vaccination monitoring, surveillance of *Trichinella* spp.). For each fox, age (juvenile: foxes < 1 year old; adult: foxes > 1 year old), gender, date and geographical location of the finding/culling were annotated.

Each fox was then georeferenced according to the procedure outlined in Obber et al. [16] (Fig. 1).

In accordance with Boitani et al. [29], each fox was assigned, on the basis of the time of finding, to one of six temporal classes of the biological cycle of the species:

- January–February: mating period;
- March–April: cubs birth period and early denning period, when cubs remain in the dens or in their immediate surroundings;
- May–June: cubs spend time and become visible also outside the dens, seeking food;
- July–August: cubs begin to be more independent;
- September–October: cubs begin to move out of their family group;
- November–December: dispersal period, when cubs move to find, establish and mark a new territory.

### Parasitological and molecular analysis

Fecal samples were kept frozen at − 80 °C for at least 72 h to inactivate eventual *Echinococcus* eggs and then tested using a filtration/sieving technique modified to enhance the likelihood to detect taeniid eggs [30]. Briefly, 2 g of feces was suspended in tap water, centrifuged and the sediment re-suspended in a zinc chloride solution and centrifuged. The supernatant was then filtered through sieves of different mesh sizes (41 µm and 21 µm) to concentrate taeniid eggs and exclude other ones. The presence of cestode eggs was verified using a bifocal inverted microscope (Leica, Wetzlar, Germany).

Cestoda eggs were collected and DNA extracted by using the DNeasy Blood & Tissue kit (Qiagen), according to the manufacturer’s instructions.

A multiplex PCR assay was performed using three couples of primers amplifying the ND1 gene for *Echinococcus multilocularis* (394 bp) and 12S rRNA for both *E. granulosus* (117 bp) and *Taenia* spp. (271 bp) [31]. Then, all the positive samples were amplified using a PCR assay targeting a fragment of cytochrome oxidase gene (460 bp) [32] and sequenced to confirm or determine the identity of the species. The PCR products were directly sequenced in a 16-capillary ABI PRISM 3130xl Genetic Analyzer (Applied Biosystems, Foster City, CA, USA), using the Big Dye Terminator v3.1 cycle sequencing kit (Applied Biosystems, Foster City, CA, USA). Sequence data were assembled and edited with SeqScape software v2.5 (Applied Biosystems, Foster City, CA, USA) and compared with representative sequences available in GenBank (https://blast.ncbi.nlm.nih.gov/Blast.cgi), using the Basic Local Alignment Search Tool (BLAST) [33] to identify the cestode species. For all PCR runs, DNA of pathogen-positive and negative samples (sterile water) served as control.

### Statistical analysis

Mapping and data spatial manipulation were carried out using ESRI ArcMap (ArcGIS Desktop: Release 10.5.1. Redlands, CA: Environmental Systems Research Institute. Copyright © 1999–2017).

To highlight the differences by age and gender, a chi-squared test (χ^2^) was performed. Pearson’s chi-squared test for linear correlation was used to test for differences over time and by ecoregion in cestode prevalence (Tables 2 and 3) and, for the most represented cestode species (*T. crassiceps, T. polyacantha* and *E. multilocularis*), to assess the independence of outcomes from sample size and distribution. The Cochran-Armitage chi-squared test for trend in proportions was performed to evaluate the presence and intensity of a linear tendency in prevalence throughout the years. To test the importance of the linear trend in the yearly prevalence variations, Pearsons’ and Cochran-Armitage were then compared and residual chi-square analyzed. Considering the importance of a consistent monitoring of *E. multilocularis*, the seasonal prevalence and its possible trend along the study period have been analyzed, both for the whole study area and within the ecoregions where this zoonotic parasite was detected. Statistical analysis was carried out using ‘stats’ package in R (R Development Core Team (2012)-URL http://www.R-project.org/).

**Table 2:** Red foxes examined by years, overall cestode prevalence

Year	Foxes tested	Positive to cestodes by coproscopy	Prevalence %
2012	1.063	67	6.30^AB^
2013	474	18	3.79^CDEF^
2014	248	19	7.66
2015	87	14	16.09^AC^
2016	227	30	13.2^BD^
2017	349	33	9.45^E^
2018	424	36	8.49^F^
Total	2.872	217	7.55

Statistically significant differences of prevalence (*p* < 0.01) are marked with equal letters

**Table 3:** Cestode positives and prevalence (%) by ecoregion

	Foxes examined	Cestode positives	Prevalence (%)
1A2a	680	32	4.70^A^
1A2b	712	74	10.39^AB^
1A2c	798	87	10.90^AC^
1B1a	26	0	0
1B1b	609	22	3.61^BC^
1D1a	47	2	4.25
Total	2.872	217	7.55

Statistically significant differences (*p* < 0.01) are marked with equal letters

## Results

Overall, 2872 fecal samples of foxes were tested from 7 provinces (Fig. 1). Samples were distributed consistently throughout the year, although peaking in late summer and autumn, in correspondence with the main hunting/control periods. According to age and gender, the sample was structured as follows: 2032 adults (71%), 533 juveniles (18%) and 307 undetermined (11%); 1169 females (41%), 1417 males (49%) and 286 undetermined (10%).

**Fig. 2:**
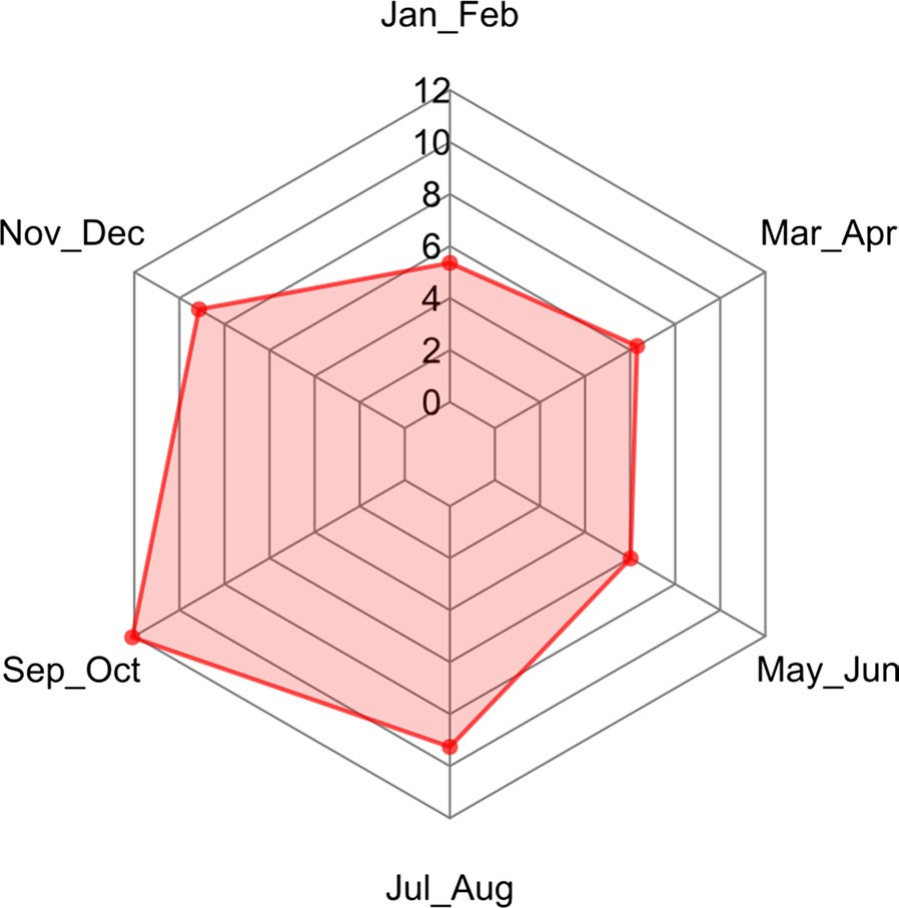
Overall cestode prevalence (%) by bimester

Two hundred seventeen samples (7.55%) tested positive for cestode eggs at coproscopy. The prevalence of cestodes as a whole was significantly higher in juveniles (12.57%) than in adults (6.25%) (χ^2^ = 22.8; *p* < 0.01), while it was almost equal among females (8.12%), males (6.84%) and undetermined (8.74%). According to sampling year, prevalence ranged from a minimum of 3.8% in 2013 to a maximum of 16.1% in 2015 (Table 2). Figure 2 shows total prevalence in foxes collected according to the bimestrial temporal classes, showing higher values in the second half of the year. Considering ecoregions, prevalence ranged from a minimum of 3.6% in 1B1b to a maximum of 10.9% in 1A2c (Table 3).

Out of the 217 positive samples at coproscopy, PCR and sequencing successfully identified 189 samples. We characterized cestodes at the species level in 175 samples and at the genus level only in other 14. Eight species were identified, with *T. crassiceps* (2.65%), *T. polyacantha* (1.98%), and *E. multilocularis* (1.04%) as the most represented. The other species, *Mesocestoides litteratus, T. krabbei, T. serialis, T. taeniaeformis* and *D. caninum*, accounted for < 0.2% each. Cestodes characterized at the genus level were identified as *Mesocestoides* spp. (11; 0.38%) and *Taenia* spp. (3; 0.10%) (Table 4). Co-infection was found in 11 foxes, which harbored *E. multilocularis* plus *T. crassiceps* (8), *T. polyacantha* (1)*, T. krabbei* (1) and *Taenia* spp. (1).

**Table 4:** Cestode species identified by DNA sequence and prevalence (%) calculated on foxes positive for cestodes (coproscopy) and on the whole fox sample

Cestode species	Positive foxes	% (*n* = 217)	% (*n* = 2.872)
*Taenia crassiceps*	76	35.0	2.65
*Taenia polyacantha*	57	26.2	1.98
*Echinococcus multilocularis*	30	13.8	1.04
*Taenia krabbei*	2	0.92	0.07
*Taenia taeniaeformis*	2	0.92	0.07
*Dipylidium caninum*	2	0.92	0.07
*Taenia serialis*	1	0.46	0.03
*Mesocestoides litteratus*	5	2.30	0.17
*Mesocestodes* spp.	11	5.06	0.38
*Taenia* spp.	3	1.38	0.10

Representative sequences of cestode species (*Taenia crassiceps, Taenia krabbei, Taenia polyacantha, Taenia taeniaeformis, Dipylidium caninum, Echinococcus multilocularis*) were submitted to GenBank (accession numbers MT806358 to MT806363).

**Fig. 3:**
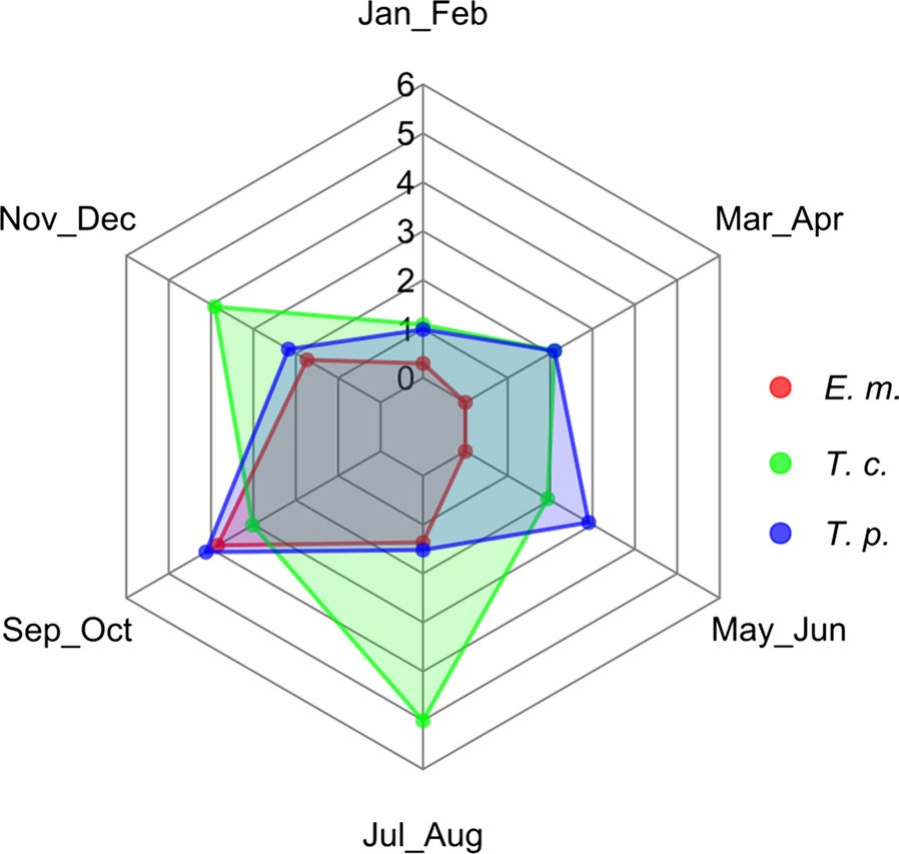
*E. multilocularis* (E. m.), *T. crassiceps* (T. c.) and *T. polyacantha* (T. p.) overall prevalence (%) by bimester

**Fig. 4:**
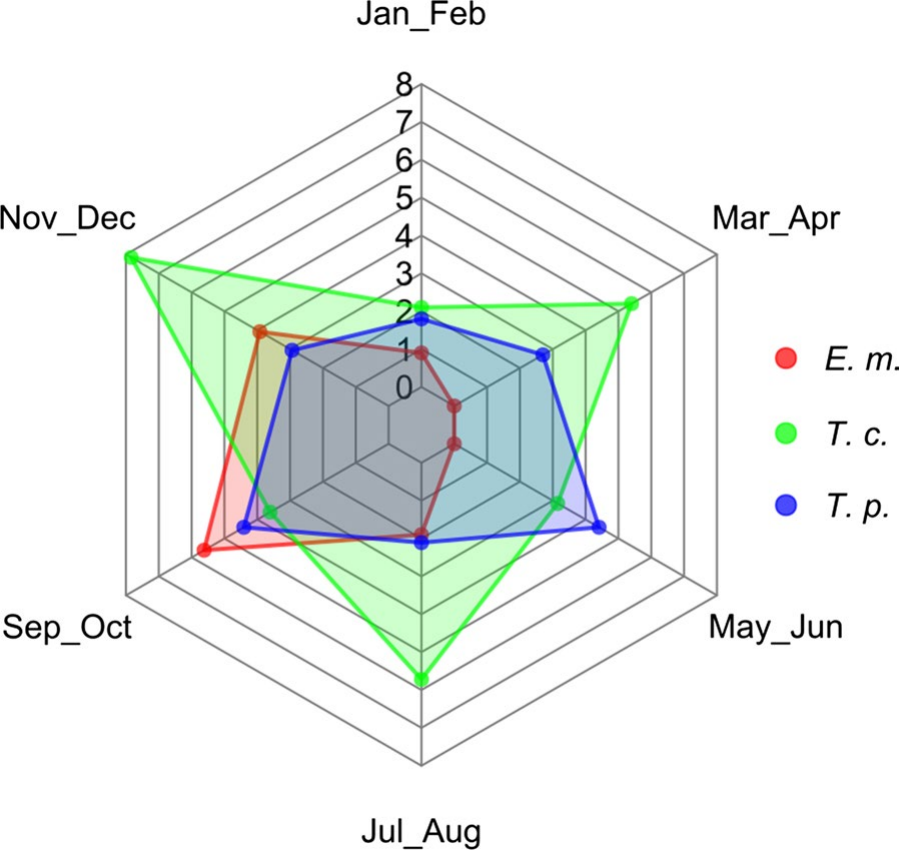
Joined *E. multilocularis* (E. m.), *T. crassiceps* (T. c.) and *T. polyacantha* (T. p.) prevalence (%) in ecoregions 1A2b and 1A2c by bimester

Considering ecoregions (Table 5), *E. multilocularis* was found only in two out of six (1A2b and 1A2c), while the other cestode species were found also in other ones. The spatial distribution of negative and positive foxes for *E. multilocularis* and cestodes is shown in Fig. 1. The shape of the distribution of main cestode species by bimester is similar to the one observed for cestodes collectively (Fig. 2), for both the whole study area (Fig. 3) and the focus ecoregions 1A2b and 1A2c (Fig. 4), also in this case showing higher values in the second half of the year.

**Table 5:** Cestode species identified by DNA sequence and relative prevalence (%) by ecoregion

Cestode species	Ecoregion					
	1A2a *n* = 680	1A2b *n* = 712	1A2c *n* = 798	1B1a *n* = 26	1B1b *n* = 609	1D1a *n* = 47
*Echinococcus multilocularis*	0	8 (1.12)	22 (2.75)	0	0	0
*Taenia crassiceps*	3 (0.44)	31 (4.35)	32 (4.01)	0	10 (1.64)	0
*Taenia polyacantha*	11 (1.62)	26 (3.65)	19 (2.38)	0	1 (0.16)	0
*Taenia krabbei*	0	0	2 (0.25)	0	0	0
*Taenia serialis*	0	0	0	0	0	1 (2.12)
*Taenia taeniaeformis*	0	2 (0.28)	0	0	0	0
*Taenia* spp.	1 (0.15)	0	2 (0.25)	0	0	0
*Mesocestoides litteratus*	3 (0.44)	0	1 (0.12)	0	1 (0.16)	0
*Mesoceistodes* spp.	2 (0.29)	3 (0.42)	6 (0.75)	0	0	0
*Dipylidium caninum*	1 (0.15)	0	1 (0.12)	0	0	0
Total	21	70	85	0	12	1

**Fig. 5:**
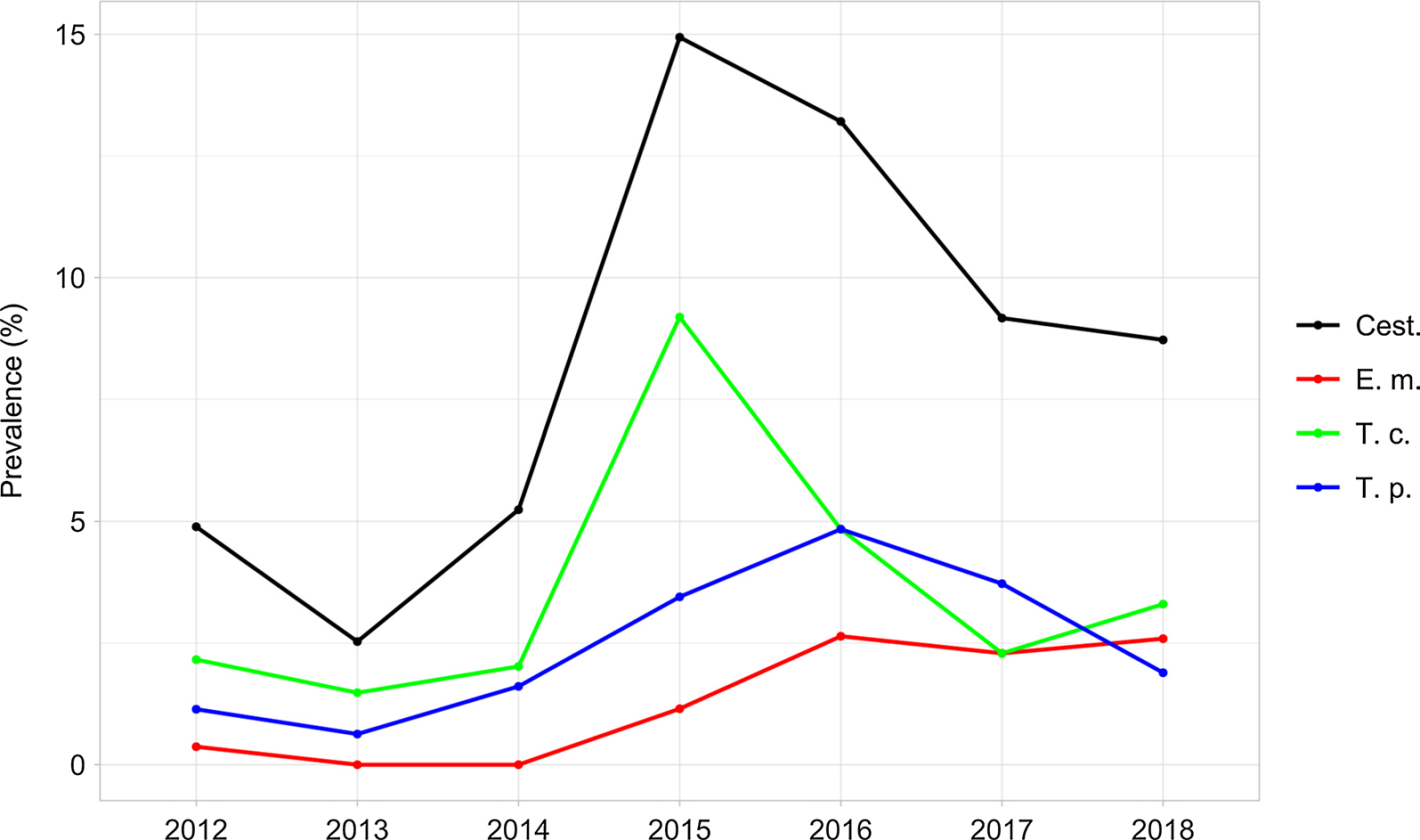
Cestodes (Cest.), *E. multilocularis* (E. m.), *T. crassiceps* (T. c.) and *T. polyacantha* (T. p.) overall prevalence (%) in the study area by year

**Fig. 6:**
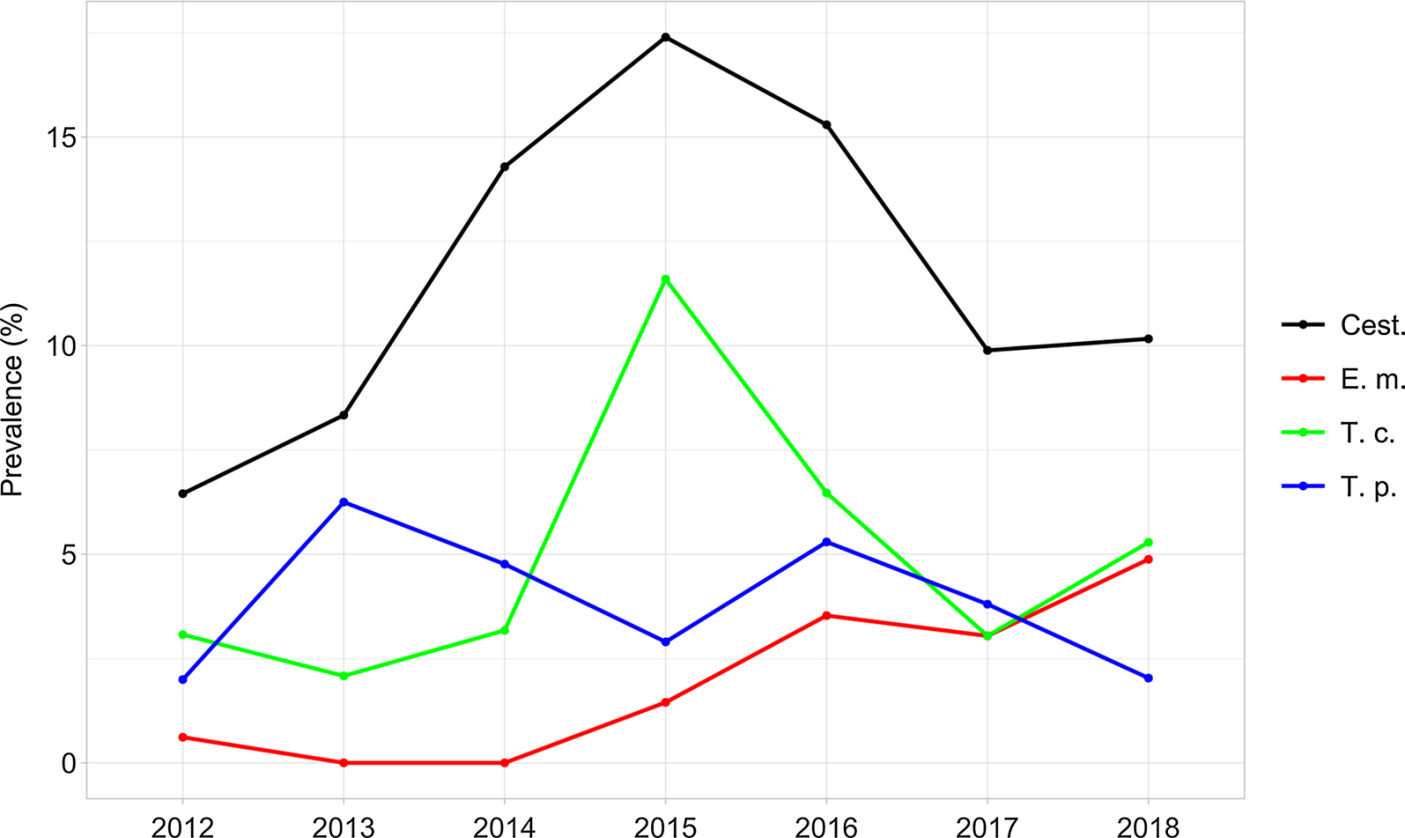
Cestodes (Cest.), *E. multilocularis* (E. m.), *T. crassiceps* (T. c.) and *T. polyacantha* (T. p.) overall prevalence (%) in ecoregion 1A2b and 1A2c by year

Prevalence variations across the years of the study were evident and statistically significant for the whole study area (Fig. 5), for both cestodes collectively (Pearson χ^2^ = 44.0; *p* < 0.01) and the three most consistent parasite species (*E. multilocularis*: Pearson χ^2^ = 32.1, *p* < 0.01; *T. crassiceps*: Pearson χ^2^ = 21.4, p < 0.01; *T. polyacantha*: Pearson χ^2^ = 21.2, *p* < 0.01). Similar results were obtained when analyzing data from the two *E. multilocularis*-affected ecoregions, with the only exception of *T. polyacantha* (All cestodes: Pearson χ^2^ = 16.5, *p* < 0.05; *E. multilocularis*: Pearson χ^2^ = 20.1, *p* < 0.01; *T. crassiceps*: Pearson χ^2^ = 14.1, *p* < 0.05; *T. polyacantha*: Pearson χ^2^ = 8.5, *p* > 0.05) (Fig. 6).

A statistically significant linear trend pattern in prevalence across the years was also evidenced on the whole study area for cestodes collectively (Armitage χ^2^ = 25.6; *p* < 0.01), *E. multilocularis* (Armitage χ^2^ = 27.1; *p* < 0.01) and *T. polyacantha* (Armitage χ^2^ = 8.1; *p* < 0.01).

Focusing the analysis on the two *E. multilocularis*-affected ecoregions, a linear trend was still statistically detectable for cestodes collectively (Armitage χ^2^ = 7.0; *p* < 0.01) and for *E. multilocularis* (Armitage χ^2^ = 19.3; *p* < 0.019). However, it must be pointed out that, both on the whole and in the focus areas, residual χ^2^ exceeded the respective critical tabulated value of χ^2^ except for *E. multilocularis*. Therefore, only for this parasite could a linear trend reasonably account for most of the inter-annual variation in prevalence.

## Discussion

Besides *E. multilocularis* and *T. krabbei*, the other species of cestodes identified in our study constitute part of the typical parasite fauna of red foxes in Italy [24, 34–38]. In particular, our results confirmed the presence of *E. multilocularis* in red foxes of northeast Italy and highlighted a tendency of increase in prevalence through the years. The focus of this zoonotic parasite seems to be restricted to the temperate semi-continental areas of the eastern Italian Alps, especially to the innermost region of the northeastern Alps subsection (ecoregion 1A2c) and the adjoining valleys of the Dolomiti and Carnia subsection (ecoregion 1A2b). In previous studies [21–23], *E. multilocularis*-positive foxes occurred close to the Austrian border, leading to the hypothesis of a transboundary focus of echinococcosis in the Alps. However, further research using a multi-locus microsatellite analysis supported the hypothesis of an autochthonous focus in Italy rather than a recent incursion of infected foxes from abroad [39, 40]. Within a fox population, the majority of animals migrating in new areas are usually juveniles, which disperse from their native territory and may also spread the parasite marking their new home range with faeces [1]. The higher prevalence of *E. multilocularis* in juveniles compared to adults, found also by other authors [41], is likely to be the reason for the seasonal distribution pattern observed in cestode prevalence: actually, in the early months of the year juveniles are absent. At the same time, this helps to identify juveniles as the main drivers of a possible expansion of the focus, since adult foxes might acquire partial immunity after repeated exposure [41].

Fox population dynamics are undoubtedly a key factor in the maintenance and possible spreading of *E. multilocularis* towards new areas but, although the red fox is managed across all the country, in Italy it is quite difficult to get robust data on this topic. Actually, the red fox is often viewed as a “pest” [42], and, as its value as a game species is considered low, no particular attention is paid to collecting and collating either hunting bags or population estimates among different areas and across the years. Besides regular hunting, wildlife managers often resort to additional numeric control measures to curtail demographic increases of the fox, in some cases also to lower predation upon endangered (e.g. white partridge, capercaillie, mountain hare), game (e.g. common pheasant, brown hare) and domestic (e.g. rabbits and poultry) species. However, even on a relatively small scale, hunting and control are often performed differently, so that their effects are not homogeneous and are difficult to measure. In fact, different studies (e.g. [43]) have shown how culling may turn out to be ineffective or even counterproductive in both fox demography and epidemiological terms, especially when applied to insufficient knowledge of ecological priors. In addition, besides hunting/control culling, in our study area at least two other important issues could have affected fox dynamics and consequently may have influenced the parasite patterns: namely, subsequent epizootics of canine distemper virus and a severe rabies epizootic from 2008 to 2011 [14, 15]. These events have caused dramatic mortality in the fox population, affecting therefore its size, dynamics, spatial distribution and home range stability. It should be noted that, while oral vaccination eradicated rabies thus stopping its spread towards the western portion of our study area, cyclic distemper epizootics in foxes have been affecting all northeastern Italy (and all the Alps) since 2006 onwards, and still occur. After dramatic perturbations as such, the fox population tends to fill the gap due to massive mortality by both new births and immigration of foxes from neighboring areas [44]: as an example, the population decrease induced by a rabies epizootic may be filled in 2 or 3 years, if human pressure remains low to moderate [29]. In such a situation, a shift of the population structure towards the juveniles might have affected the fox parasite community, including *E. multilocularis* and other cestodes as the most represented *T. crassiceps*, as well as their apparent upsurge in 2015–2016.

Of course, the definitive host population represents only one of the ecological determinants of the parasite community. Among the others, spatial and temporal variations in the composition and abundance of the intermediate host species community play a paramount role: differences in prevalence compared to other Italian and European regions are likely to be modulated by the distribution and abundance of the local intermediate hosts [45–47]. In our research, *E. multilocularis* and *T. crassiceps* were more frequently observed in the second half of the year (Fig. 3) probably because population densities of voles are higher in this period [47].

The diet of the red fox in the Alpine area includes small and medium sized mammals [11, 12], which represent intermediate hosts for larval stages of cestodes, but opportunistic carnivores such as foxes can easily adapt their home range size and food habits to resource availability [48]. To this purpose, the increase in ungulate populations observed in the last few decades has probably represented an important factor not only for the re-colonization by large carnivores in Europe [49], but also for the abundance of medium-sized carnivores such as foxes especially where, as has been occurring in the Alps, many human activities have been abandoned [50]. The presence of *Taenia krabbei,* reported in the Arctic fox [45, 46, 51], common in wolves in Italy [26, 52, 53] but rarely observed in the red fox [54], can be related to higher frequency of consumption of ungulate carcases by foxes. For this purpose, it is interesting to point out that, in recent years, cysticercosis due to *T. krabbei* has been indentified for the first time in Italy in two hunted roe deer (*Capreolus capreolus*) and a dog of the Italian northern Apennines [55]. A follow-up of the prevalence of this cestode species in our country could give further information on its ecology and taxonomic status, as mitochondrial DNA analyses of morphologically similar isolates from northern Europe revealed the presence of cryptic species [56]. In our study area, however, few data are available assessing the feeding habits of red foxes, mainly from scat analysis [11, 12]. As an example, in the Fiemme Valley fruits were observed as the main trophic resource in the diet of the red fox; insects, mainly ground-living Coleoptera, and roe deer (*Capreolus capreolus*) were a secondary food source, while rodents were rarely found [12]. Notwithstanding, apart from *D. caninum* and *T. krabbei*, which recognize respectively fleas and large ungulates as intermediate hosts, all other cestode species found in our study have rodents as intermediate hosts. Therefore, it is presumable that these small mammals still represent a consistent component of the diet of the red fox in northeast Italy, although less important than a time [11, 12, 38]. It is understood, however, that the intensity of predation on small rodents, as well as small rodent species' community abundance and composition, can vary according to the ecological context and the opportunity for foxes to exploit different food sources. Such a variability could at least partially account for the limited geographical range observed for *E. multilocularis*, for which not all rodent species have the same competence [57], but the ecoregions as described appear drawn at a scale too large to properly investigate this question.

Focusing on the main object of this study, *E. multilocularis*, the increasing trend evidenced may be partially due to a likewise increasing attention paid to this zoonotic parasite over the years: however, although there is no doubt about this increasing interest, in the years of our study the methods have been kept consistent.

Compared to previous studies, its mean prevalence in the Bolzano territory (2.8%) is substantially lower. Indeed, in red foxes collected from 2001 to 2005 and tested through PCR, *E. multilocularis* was found in 12.9% of foxes from Bolzano and 2.9% of foxes from Trento provinces [21]. On the contrary, we did not find positive foxes in Trento province, and the prevalence of *E. multilocularis* even in the most affected ecoregion (1A2c) was 2.75%. Prevalence determined by PCR on cestode eggs isolated from the faeces (egg-PCR) is likely to be underestimated: Otero-Abad et al. [58] calculated a good specificity of 93.4% and a moderate sensitivity of 54.8% for the same egg-PCR technique we used. Our prevalence of *E. multilocularis* is very similar to the 2.6% found in Slovenia in 2010 [17], but it is much lower than that recorded in endemic areas of Europe. There, *E. multilocularis* may reach a very high prevalence in red foxes locally, such as 28.5% in Denmark [59] and 25.6% in Poland [60], and in general a prevalence > 10% in eastern countries (reviewed in Oksanen [3]), where human cases are annually reported [61]. In such areas, due to the high prevalence of the parasite, the positive predictive value of diagnostic tests will probably be much higher than in our territory, in which *E. multilocularis* seems instead a patchy and rare pathogen with very low prevalence. Therefore, estimates in areas as wide as provinces/regions/groups of regions (as the whole northeastern Italy) should be considered with particular caution, all the more reason considering that on such a scale a wide variability among the yearly sample sizes seems unavoidable. In our context, higher prevalence values can be found at a very local level (e.g. the Alta Val d’Isarco district, an area covering about 650 km^2^, where prevalence reaches 11%), and in the absence of specific and local models to predict the potential reach of the parasite, we suggest results to be presented on a scale determined by ecological constraints.

Italy has never officially reported a confirmed autochthonous case in humans, but at the end of the nineteenth century, human cases were recorded in Pusteria valley and from Bressanone, both in Bolzano province ([62], cited in 21). The incidence of human cases of *E. multilocularis* in Europe is approximately proportional to the prevalence in the definitive hosts and showed a significant increasing trend in the years 2008–2016 [61]. In endemic areas, the increase of fox density close to villages may put dogs at risk of infection and consequently increase the risk for humans. Indeed, in an endemic area of France, *E. multilocularis* was detected by PCR in 35%, 11% and 7% of fox, dog and cat feces, respectively, collected in kitchen gardens [63]. Whenever the increasing trend that we observed is confirmed and persists, an increased risk for humans will follow, also in our focus area. As a final remark, among the species of cestodes found in our study, some have been designated zoonotic as well, i.e. *T. crassiceps*, *T. taeniaeformis* and *T. serialis*, causing cysticercosis, strobilocercosis and coenurosis, respectively [64]. However, cases in humans are rare worldwide and do not represent a major public health concern.

## Conclusions

Our study confirms the presence of a focus of *E. multilocularis* in red foxes of northeastern Italy, apparently still confined in its northwestern portion. Given the persistence of the focus through the years, and the apparent increase in prevalence during our study, we recommend an informative campaign on how to behave to avoid infection at a local level, targeted at people living in the area, especially hunters, dog owners, forestry workers and other groups at risk. In Italy, *E. multilocularis* in foxes, as well as alveolar echinococcosis in intermediate hosts, including humans, should be approached as rare diseases. Consequently, rather than screening on a large scale, further work should focus on the ecology of this disease in the definitive and intermediate hosts and in the environment on a small scale in the areas where its presence has already been demonstrated, with the final aim to understand which are the factors that allow the persistence and possible spreading of this parasite. For this purpose, research is in progress to test more sensitive diagnostic methods and samples (e.g. from necropsies) in definitive and intermediate hosts to detect other possible foci as well as to address the role of density and dispersal pathways of foxes and small mammal community composition in the maintenance and spreading of this severe zoonosis.

## Data Availability

All data generated or analyzed during this study are included in this published article.
